# The risk factors for the failure of hook wire localization of ground glass nodules prior to thoracoscopic surgery

**DOI:** 10.1186/s13019-022-01866-y

**Published:** 2022-05-11

**Authors:** Musu Ala, Junzhong Liu, Jieli Kou, Xinhua Wang, Minfeng Sun, Changcheng Hao, Jianlin Wu

**Affiliations:** 1 Graduate School, Tianjin Medical University, Tianjin, 300070 People’s Republic of China; 2 Department of Ultrasound, The Affiliated Hospital of Inner Mongolia Medial University, Hohhot, Inner Mongolia 010050 People’s Republic of China; 3 Department of Radiology, Weifang People’s Hospital, The First Affiliated Hospital of Weifang Medical University, Weifang, 261041 Shandong People’s Republic of China; 4 Department of Medical Imaging, Cangzhou People’s Hospital, Cangzhou, 061001 Hebei People’s Republic of China; 5 Department of Radiology, Weifang No. 2 People’s Hospital, The Second Affiliated Hospital of Weifang Medical University, Weifang, 261041 Shandong People’s Republic of China; 6 Thoracic Surgery Department, Weifang No. 2 People’s Hospital, The Second Affiliated Hospital of Weifang Medical University, Weifang, 261041 Shandong People’s Republic of China; 7 Department of Medical Imaging, Affiliated Zhongshan Hospital of Dalian University, 6 Jiefang Street, Dalian, 116001 Liaoning People’s Republic of China

**Keywords:** Pneumothorax, Localization, Ground glass nodule, Thoracoscopic surgery

## Abstract

**Objectives:**

To retrospectively analyse the potential influencing factors of CT-guided hook wire localization failure prior to thoracoscopic resection surgery of ground glass nodules (GGNs), and determine the main risk elements for localization failure.

**Methods:**

In all, 372 patients were included in this study, with 21 patients showing localization failure. The related parameters of patients, GGNs, and localization were analysed through univariate and multiple logistic regression analysis to determine the risk factors of localization failure.

**Results:**

Univariate logistic regression analysis indicated that trans-fissure (odds ratio [OR] 4.896, 95% confidence interval [CI] 1.489–13.939); trans-emphysema (OR 3.538, 95% CI 1.343–8.827); localization time (OR 0.956, 95% CI 0.898–1.019); multi-nodule localization (OR 2.597, 95% CI 1.050–6.361); and pneumothorax (OR 10.326, 95% CI 3.414–44.684) were risk factors for localization failure, and the p-values of these factors were < 0.05. However, according to the results of multivariate analysis, pneumothorax (OR 5.998, 95% CI 1.680–28.342) was an exclusive risk factor for the failure of preoperative localization of GGNs.

**Conclusion:**

CT-guided hook wire localization of GGNs prior to thoracoscopic surgery is often known to fail; however, the incidence is low. Pneumothorax is an independent risk factor for failure in the localization process.

## Background

Early resection of pulmonary ground glass nodules (GGNs) with malignant signs can improve the survival rate of patients [1]. GGN is a nodule that radiologically opaque with underlying opacity of pulmonary blood vessels or bronchial structures, which is closely related to early adenocarcinoma. Video-assisted thoracoscopic surgery (VATS) is a recognized and effective option to resect malignant GGNs early, and has been widely applied given that it is less invasive than traditional open thoracotomy [2–4]. Because of the soft texture of GGNs, it is difficult for thoracic surgeons to touch the nodules with their fingers [5]. Therefore, it is necessary to locate the hook wire marker under the guidance of computed tomography (CT) to achieve precise localization and resection of GGNs through VATS [5, 6].

Hook wire is one of the most commonly used preoperative markers that has a high success rate [4, 7, 8]. However, there are still risks of localization failure, such as dislodgement or displacement [9, 10], which likely affect the precise removal of pulmonary nodules in thoracoscopic surgery. Despite this, there are few reports on the failure of preoperative localization of pulmonary nodules [11, 12].

In this paper, we retrospectively analysed the potential risk factors of failure to localize the GGNs under CT guidance prior to VATS and determined predictors for localization failure.

## Methods and materials

All patients signed the informed consent before CT-guided localization of GGNs. Because all the data are anonymous, our institutional review board allowed this retrospective study and waived the need for informed consent to collect and use the patients’ data.

### Patients

**Fig. 1:**
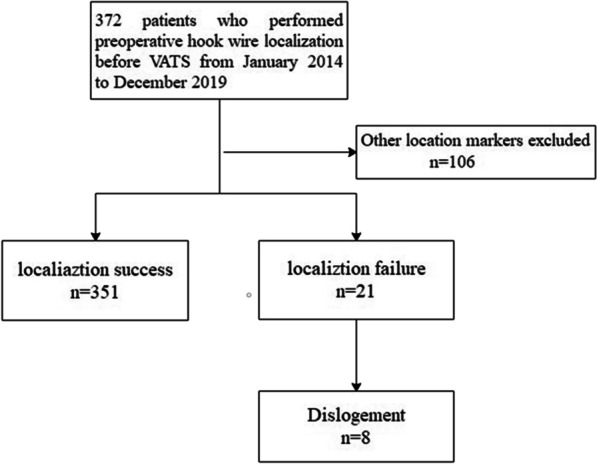
Flowchart of the study population

From January 2014 to December 2019, a total of 372 patients (single nodules, n = 351; multiple nodules, n = 21) who underwent preoperative hook wire localization before VATS surgery were enrolled in this study. The inclusion criteria were as follows: (1) simultaneous localization of single or multiple GGNs in unilateral lung; (2) hook wire localization; (3) subpleural depth of GGNs > 5 mm and/or < 40 mm. The exclusion criteria were as follows: (1) depth of GGNs > 40 mm or < 5 mm; (2) bilateral pulmonary nodules localized simultaneously; (3) large blood vessels or bronchi around or across the nodules; and (4) other localization markers. The enrolled patients were divided into the success and failure groups. The flowchart of the study population is exhibited presented in Fig. 1.

### Hook wire localization

An attending radiologist with five years’ experience completed the process of localization of GGNs in collaboration with the thoracic surgeon. The localization set of the hook wire was 20G × 120 mm (Pajunk, Germany). The 64-slice spiral CT used for preoperative localization was manufactured by GE Healthcare (Connecticut State, USA).

**Fig. 2:**
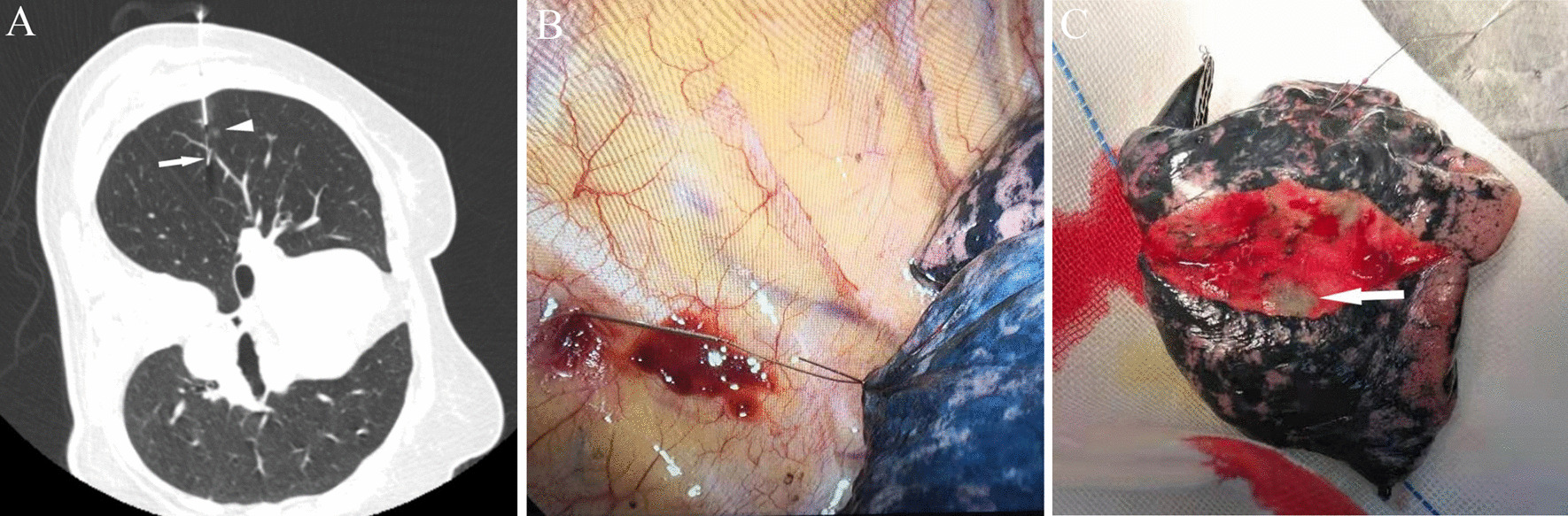
58-year-old woman who underwent hook wire localization prior to VATS. **a** Hook wire (white arrow) was positioned next to the ground glass nodule (white triangle arrow) of the right upper lobe before thoracoscopic surgery. **b** Hook wire was seen on the pleura surface during thoracoscopic surgery. **c** The main pathology of the lesion after thoracoscopic resection (the white arrow shown the ground glass nodule)

After ensuring the appropriate body position and localization path based on the previous CT images, the patient underwent preoperative marker localization process of GGNs under local anaesthesia along the shortest localization pathway as far as possible. Once the tip of the guide needle reached around the target nodule, the hook wire was released and the guide needle withdrawn. If the needle tip did not reach the nodule position, the guide needle was re-adjusted to the ideal position. The ideal position is to locate the hook wire around or cross the target nodule within 1 cm. After withdrawal of the guide needle, the CT is repeated to confirm whether the localization is successful (Fig. 2a). If the localization operation is successful, the patient will be sent to the operating room accompanied by a nurse within 1 h. However, if the procedure fails by way of dislodgement or displacement, the localization operation will be retried with the patient’s permission, regardless of whether the localization is successful. The results of localization need to be relayed to the thoracic surgeon, so that the surgical method can be determined according to the situation.

### VATS

The patients underwent dual-port thoracoscopic resection in a suitable position under general anaesthesia. The VATS operation is performed by visualizing the end of the hook wire on the pleural surface under the guidance of nodule localization (Fig. 2b). The choice of lobectomy, wedge resection or segment resection is made by the thoracic surgeon. Next, the resected specimens are sent to the pathology department for a quick frozen-section examination to confirm whether the resected lesions are benign or malignant (Fig. 2c). Hilar and mediastinal lymph nodes are usually dissected, and the nature of the nodes are confirmed pathologically.

### Data collection and variables definition

The baseline information of all patients was collected by an attending radiologist through the Picture Archiving and Communication Systems, and the imaging data was measured and recorded on the GE Advantage Workstation (v.4.6). The baseline variables include the patient’s age, sex, and smoking status. The parameters of GGNs are as follows: diameter; depth of GGN (in millimetres from the target nodule to the nearest pleura [the depth is recorded according to the shallowest nodule when multiple GGNs are localized]); nodule position (upper and middle or lower lobe); and whether multiple GGNs are localized. The localization-related variables are puncture position (supine, prone, or lateral); trans-emphysema (in the localization pathway); trans-fissure (transfissural approach in the localization pathway); location time; pneumothorax; pulmonary haemorrhage (localization related); and localization result (success or failure).

The definition of placement failure is as follows: (1) The hook wire marker could not be successfully released during the localization process; (2) although the hook wire was successfully placed on the target nodule for the first time or once again, the dislodgement or decoupling occurred during CT confirmation after the localization; (3) preoperative localization of the hook wire was accurate and successful under the CT guidance, but the marker was found to fall off during thoracoscopic surgery.

### Statistical analysis

All 372 patients were divided into two groups according to whether the localization was successful, and the risk factors of localization failure were assessed. The variable factors that were established to be different in the univariate logistic regression analysis were incorporated into the multiple logistic regression analysis to identify the determinants of the localization failure. The diagnostic ratio (OR) and 95% confidence interval (CI) of each independent factor was calculated. Further, p < 0.05 was considered to indicate statistical significance. SPSS software (v.25, IBM Corporation, Armonk, NY, USA) was utilized for statistical analyses.

## Results

The baseline characteristics of the participants and parameters related to localization are shown in Table 1. Among the 372 patients who were localized before VATS, 351 patients were successfully localized, and the other 21 cases showed failure, of which eight cases were attributed to dislodgement. When repeating the CT scan to confirm the position after localization, it was found that hook wires protruded from the lung parenchyma and were accompanied by pneumothorax in five out of eight cases. The other three cases showed dislodgement and pneumothorax during thoracoscopic surgery after being sent to the operating room after successful preoperative localization. The reason for the remaining 13 cases of failure was that pneumothorax occurred during the localization process and could not be accurately localized, although relocation was attempted. For the cases that could not be located, segmental resections were done by the hematoma on the pleural surface in three cases, and the remaining cases underwent lobectomy under VATS.

**Table 1:** The baseline characteristics of the participants and parameters related to localization

Variable	Success (n = 351)	Failure (n = 21)	P value
Age	58.95 ± 9.96	59.62 ± 10.33	0.764
*Sex*			0.825
Male	179 (51.0%)	10 (47.6%)	
Female	172 (49.0%)	11 (52.4%)	
*Smoke*			0.532
No	256 (72.9%)	14(66.7%)	
Yes	95 (27.1%)	7 (33.3%)	
*Localization of multiple nodules*			0.030
No	260 (74.1%)	11 (52.4%)	
Yes	91 (25.9%)	10 (47.6%)	
Localization time (min)	22.20 ± 10.52	19.14 ± 7.16	0.190
Depth of nodule (mm)	21.34 ± 9.17	15.52 ± 6.85	0.005
Diameter (mm)	8.99 ± 3.80	9.29 ± 3.47	0.732
*Trans-fissure*			0.002
No	329(94.0%)	16 (72.2%)	
Yes	21 (6.0%)	5 (23.8%)	
*Trans-emphysema*			0.005
No	299 (85.2%)	13 (14.8%)	
Yes	52 (69.9%)	8 (38.1%)	
*Puncture position*			0.450
Supine	112 (31.9%)	9 (42.9%)	
Prone	153 (43.6%)	9 (42.9%)	
Lateral	86(24.5)	3(14.3%)	
*Location*			0.737
Upper and middle	197 (56.1%)	11 (52.4%)	
lower	154 (43.9%)	10 (47.6%)	
*Pneumothorax*			< 0.001
No	222(63.2%)	3(14.3%)	
Yes	129(36.8%)	18(85.7%)	
*Pulmonary hemorrhage*			0.075
No	301(85.8%)	15(71.4%)	
Yes	50(14.2%)	6(28.6%)	

Pneumothorax occurred in 39.52% (147/372) of all localization patients, and the rate in the failure group was 87.5% (18/21) during the CT-guided localization process. Univariate logistic regression analysis indicated that trans-fissure (OR 4.896, 95% CI 1.489–13.939); trans-emphysema (OR 3.538, 95% CI 1.343–8.827); localization time (OR 0.956, 95% CI 0.898–1.019): multi-nodule localization (OR 2.597, 95% CI 1.050–6.361); and pneumothorax (OR 10.326, 95% CI 3.414–44.684) were risk factors for localization failure, and the *p*-values of these factors were < 0.05. However, according to the results of multivariate analysis, pneumothorax was an exclusive risk factor for the failure of preoperative localization of GGNs. The results of univariate and multivariate regression analysis are presented in Table 2.

**Table 2:** Univariate and multivariable analysis of baseline and CT features

	Univariable analysis			Multivariable analysis		
	OR	95% CI	P value	OR	95% CI	P value
Age	1.007	0.963–1.053	0.763			
*Sex*						
Male	1.0					
Female	1.145	0.471–2.814	0.764			
*Smoke*						
No	1.0					
Yes	1.347	0.528–3.440	0.533			
*Localization of multiple nodules*						
No	1.0					
Yes	2.597	1.050–6.361	0.035	1.346	0.494–3.595	0.552
Location time (min)	0.956	0.898–1.019	0.166			
Depth of nodule (mm)	0.910	0.850–0.973	0.006	0.942	0.875–1.014	0.113
*Trans-fissure*						
No	1.0					
Yes	4.896	1.489–13.939	0.005	2.168	0.613–6.773	0.199
*Trans-emphysema*						
No	1.0					
Yes	3.538	1.343–8.827	0.008	1.003	0.343–2.806	0.995
Diameter (mm)	1.020	0.912–1.141	0.731			
*Insertion position*						
Supine	1.0					
Prone	2.304	0.605–8.767	0.221			
lateral	1.686	0.445–6.396	0.442			
*Location*						
Upper and middle	1.0					
Lower	0.860	0.356–2.077	0.737			
*Pneumothorax*						
No	1.0					
Yes	10.326	3.414–44.684	< 0.001	5.998	1.680–28.342	0.010
*Pulmonary hemorrhage*						
No	1.0					
Yes	1.552	0.909–2.497	0.083			

## Discussion

A variety of preoperative markers for GGNs prior to VATS have been reported, including hook wire [4, 13], coil [14, 15], methyl blue [16], 99mTc [17], hydrogel plugs [18], barium [8, 9], and fiducial marker [8, 19]. Because of its high feasibility and usability, hook wire is the most widely used marker in preoperative localization. Although the preoperative hook wire localization has a high success rate, the reported probability of hook wire localization failure is 7.5% owing to migration or dislodgement [4, 9]. At present, there are few articles on the analysis of localization failure [11]. Therefore, the purpose of this article was to clarify the factors of localization failure to provide clinical decision-making in advance.

In this study, 21 out of 372 patients showed localization failure with an incidence rate of 5.65%, which is similar to that reported in the literature [9]. Among the failed cases, 13 developed pneumothorax during the positioning process. As the pneumothorax progresses, the tip of the guide wire protrudes out of the lung parenchyma, and the hook wire cannot be released to the target nodule. Among the 21 failed cases, 13 showed pneumothorax before the guide wire was released successfully during the localization process. As the pneumothorax progresses, the guide wire tip protrudes out of the pleural cavity and the hook wire cannot be released to the target nodule. Even if the positioning is reinitiated, it is difficult to fix the guide needle to the ideal position within the calculated distance given the pneumothorax, which causes the swing of the pleural cavity. Usually, when pneumothorax occurs, the depth of localization is longer than the predetermined distance given the retraction of the lung tissue. However, it is difficult for the doctor to insert the guide needle excessively beyond the predetermined distance during operation to avoid damage to large blood vessels and cause severe haemoptysis. The remaining eight failed cases of dislodgement were found with pneumothorax during CT reconfirmation (five cases) and VATS (three cases) after the initial successful localization.

In our study, the incidence of pneumothorax formation is 39.52%, which is within the range of 12.8–68.1% reported in the literature [11]. In this study, 85.7% of localization failure cases were related to pneumothorax (P < 0.001 compared with the control group). The results of multiple regression analysis also confirmed that pneumothorax was the independent factor related to localization failure. Literature reports show that trans-fissure is an important influencing factor of localization failure [10, 11]. In our results, trans-fissure showed a significant difference in the univariate logistic analysis. It is an important influencing factor rather than an independent factor, which is different from the literature reports. Because the trans-fissure approach has to penetrate three layers of pleura, the incidence of pneumothorax with the trans-fissure approach is higher than that with the traditional approach. We speculate that the trans-fissure approach is due to the occurrence of pneumothorax and leads to localization failure; hence, the trans-fissure is a risk factor for pneumothorax, not a direct risk factor for localization failure.

According to the results of univariate logistic regression, other factors such as depth of the nodule, trans-emphysema, and localization of multiple nodules are also related to the failure of localization. The shallower the location depth, the greater the risk of dislodgment after the occurrence of pneumothorax, because the tip of the hook wire is easily pulled out of the pulmonary parenchyma under the swing of the chest cavity. Previous studies have shown that the shallower depth threshold for hook wire placement is 1.8 cm [10], so as to possibly avoid pneumothorax. In our research, the localization depth of the failure group was shallower than that of the success group (15.52 ± 6.85 *vs*. 21.34 ± 9.17, p < 0.001). Although published studies have reported that it is safe and feasible to locate multiple nodules at the same time [20], our study found that the rate of failure was higher than that of the single nodule group (47.6% vs. 25.9%, p = 0.030). Simultaneous localization of multiple nodules is related to localization failure, which may increase the risk of localization failure. Emphysema, especially trans-emphysema or pulmonary bullae in the puncture path, is the influencing factor of localization failure according to the results of our univariate logistic regression analysis. Therefore, the pre-localization plan should attempt to avoid emphysema in the lung parenchyma.

Other reported factors of localization are the location of the nodule in the lower lobe [21], surgical history [21], vital capacity [11, 22], physician experience [11], and location time [10, 23]. These factors are mainly related to the formation of pneumothorax and whether they are related to localization failure still requires further investigation. The results of multiple regression analysis in this study indicate that only pneumothorax is an independent risk factor. This could be because the tail of the hook wire is fixed on the chest wall and the hook tip that is placed on the target nodule can move relative to each other with the formation and progression of pneumothorax. The formation of pneumothorax can drag the hook wire that has been fixed to the target nodule. The hook wire tip may be displaced or pulled off into the chest cavity, resulting in failure of localization.

The limitation of this article is that the sample size of the failure group is quite small, which maybe affect the results of statistical analysis, and there is no comparison and inclusion of other commonly used metal markers to analyse the causes of failure, such as Coil.

In conclusion, although the preoperative localization success rate of GGNs under CT guidance is high, localization failure still exists. Pneumothorax is an independent risk factor for failure in the localization process.

## Data Availability

The datasets used and/or analyzed during the current study are available from the corresponding author on reasonable request.

## Abbreviations

GGN: Ground glass nodule
CI: Confidence interval
OR: Odds ratio
VATS: Video assisted thoracoscopic surgery
CT: Computed tomography
